# A Conceptual Mathematical Model of the Dynamic Self-Organisation of Distinct Cellular Organelles

**DOI:** 10.1371/journal.pone.0008295

**Published:** 2009-12-30

**Authors:** Bernd Binder, Andrean Goede, Nikolaus Berndt, Hermann-Georg Holzhütter

**Affiliations:** Institute of Biochemistry, Charité-Universitätsmedizin Berlin, Berlin, Germany; University of Arizona, United States of America

## Abstract

Formation, degradation and renewal of cellular organelles is a dynamic process based on permanent budding, fusion and inter-organelle traffic of vesicles. These processes include many regulatory proteins such as SNAREs, Rabs and coats. Given this complex machinery, a controversially debated issue is the definition of a minimal set of generic mechanisms necessary to enable the self-organization of organelles differing in number, size and chemical composition. We present a conceptual mathematical model of dynamic organelle formation based on interacting vesicles which carry different types of fusogenic proteins (FP) playing the role of characteristic marker proteins. Our simulations (ODEs) show that a *de novo* formation of non-identical organelles, each accumulating a different type of FP, requires a certain degree of disproportionation of FPs during budding. More importantly however, the fusion kinetics must indispensably exhibit positive cooperativity among these FPs, particularly for the formation of larger organelles. We compared different types of cooperativity: sequential alignment of corresponding FPs on opposite vesicle/organelles during fusion and pre-formation of FP-aggregates (equivalent, e.g., to SNARE clusters) prior to fusion described by Hill kinetics. This showed that the average organelle size in the system is much more sensitive to the disproportionation strength of FPs during budding if the vesicular transport system gets along with a fusion mechanism based on sequential alignments of FPs. Therefore, pre-formation of FP aggregates within the membranes prior to fusion introduce robustness with respect to organelle size. Our findings provide a plausible explanation for the evolution of a relatively large number of molecules to confer specificity on the fusion machinery compared to the relatively small number involved in the budding process. Moreover, we could speculate that a specific cooperativity which may be described by Hill kinetics (aggregates or Rab/SNARE complex formation) is suitable if maturation/identity switching of organelles play a role (bistability).

## Introduction

Eukaryotic cells are composed of morphologically and functionally distinct organelles such as the nuclei, mitochondria or lysosomes. Cellular organelles differ in number, size distribution, intracellular location and chemical composition. These organelle-specific features are maintained in a dynamic process in which organelles continuously exchange their biochemical material either through the uptake and release of smaller vesicles or direct fusion and budding [1]. The rate of these dynamic processes may vary thus giving rise to changes in the number, size distribution and intracellular location of organelles [2]. For example, the number of lysosomes in resting and activated macrophages may differ by more than a factor of 3 [3]. This increase in the number of lysosomes is accomplished by a higher export of lysosome-type vesicles from the endoplasmic reticulum [4]. An increase of cellular calcium may cause an enhanced fusion of vesicles [5] with the cell membrane releasing their cargo either into the external space (e.g. neurotransmitters) or inserting proteins into the cell membrane (e.g. glucose transporter).

Notably, fusion and budding processes among vesicles and organelles involve a large machinery of auxiliary proteins. In budding, induction of membrane curvature is facilitated by so-called coat proteins as clathrin, COP-I, COP-II. Membrane receptor bind a specific repertoire of organelle proteins and thus determine the protein content of released vesicles. In fusion, surface proteins like SNAREs (N-ethylmaleimide-sensitive fusion protein) are important membrane-tethering factors establishing a stable cross-link between fusing membranes. Their activation is controlled by small GTPases like Rab proteins of which more than 60 different isoforms have been identified so far [6]. Activation of Rab proteins is accomplished by the exchange of GDP/GTP catalysed by specific GEFs (GTP/GDP-exchange factors) whereas inactivation is brought about by specific GAPs (GTPase-activating proteins) catalysing the hydrolysis of GTP to GDP. The activity of these main components of the budding and fusion machinery is modulated by many other proteins functioning as co-factors and scaffolds (for excellent overviews see [7]–[10]).

As often in biochemistry, the sheer overwhelming molecular complexity of a cellular sub-system, e.g. signalling or metabolic pathway, makes it difficult to decipher the basic principles governing its design and dynamics. Mathematical models that deliberately refrain from the incorporation of all known molecular details but instead merge many molecular components to a few basic functional units may help to distil such principles (Committee on Defining and Advancing the Conceptual Basis of Biological Sciences in the 21st Century, 2008). In our understanding, approaches based on conceptual mathematical models are quite feasible considering that the sophisticated network of molecular interactions observed in present-day cells has been developed in natural evolution with the primary goal to make fundamental processes already present in primordial cells more efficient and specific. Our modelling approach addresses two cardinal questions susceptible to computational investigation: How does the kinetics of vesicle fusion and budding determine the formation of organelles of different size and how does the kinetics of complex formation between organelle-specific fusogenic proteins (e.g. SNAREs or Rabs) influence the formation of organelles with distinct biochemical identity? A pioneering computational analysis of [12] revealed that the affinity constants with which organelle-specific SNARE proteins are loaded into budding vesicles have to be sufficiently different in order to maintain the organelle identity. However, the model used in [12] is based on two pre-defined compartments and additional small unit vesicles but does not take into account the dynamic *de novo* self-organisation of organelles by allowing inter-vesicle fusion events. Hence, including these processes in our extended model and starting with an empty vesicular system, we again ask for the necessary conditions the budding and fusion machinery have to fulfil to enable the dynamic *de novo* self-organisation of biochemically diverse organelles.

In this paper, development and simulation of the model comprises two subsequent steps. First, we establish a model that describes the dynamics of a system of pure lipid vesicles that are permanently synthesised and degraded and which may undergo fusion and budding processes thereby forming larger organelle-like aggregates (L-model). We demonstrate that this model is able to reproduce experimental data on changes in the size-distribution of vesicles elicited by changes of the fusion rate. Next, we extend the L-model by including two different sorts of membrane proteins affecting the fusion and budding process (LP-model). We do not model explicitly SNAREs and Rab proteins. In our model a specific active Rab together with its specific activated SNARE and other associated effectors and characteristic resident proteins form a functional unit which we call fusogenic protein (FP). Thus, such an entity represents a lumped fusogenic protein complex playing the role of an organelle specifier or marker. Thus, the dominant presence of one type of FP defines a characteristic organelle type. Rab5 and Rab7 together with their effectors (e.g. SNAREs) may be regarded as examples of such specifiers for early and late endosomes, respectively [36].

Model simulations demonstrate that the formation and maintenance of specific organelles comprising significantly different distributions of the two membrane proteins require both a certain degree of disproportionation of the two proteins during the budding off of small unit size vesicles and a positive-cooperative kinetics of homotypic protein-protein interactions during membrane fusion. An initially disproportionated distribution of FPs into unit vesicles during budding or *de novo* generation gets lost during the formation of larger organelles assembled via permanent fusion of these vesicles if no cooperativity governs the fusion processes.

The latter finding is in line with experimental findings demonstrating the formation of a supramolecular SNARE assembly to precede membrane fusion [13]. According to our results, positive-cooperative interactions among homotypic fusion proteins appears to be not only a means to accelerate the fusions process but, more importantly, a necessary prerequisite for maintaining the biochemical identity of cellular organelles.

## Results

### Formation of Organelles from Pure Lipid Vesicles (L-Model)

First, we developed a simple mathematical model to describe the process of organelle formation in a system of interacting lipid vesicles which are homogeneously composed of a single type of membrane lipid. Our system is defined by 4 basic processes: (1) formation of “unit vesicles” (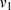) with constant rate 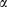, (2) degradation of unit vesicles in a first-order process proceeding with rate constant 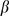, (3) fusion of arbitrary vesicles 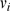 and 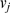 with the fusion rate 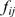 depending on the size of the vesicles and (4) decay of vesicles 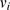 (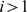) into smaller vesicles 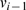 and 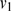, i.e. by budding off single unit size vesicles, with the budding rate 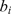 depending only on the size of 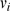.

**Figure 1:**
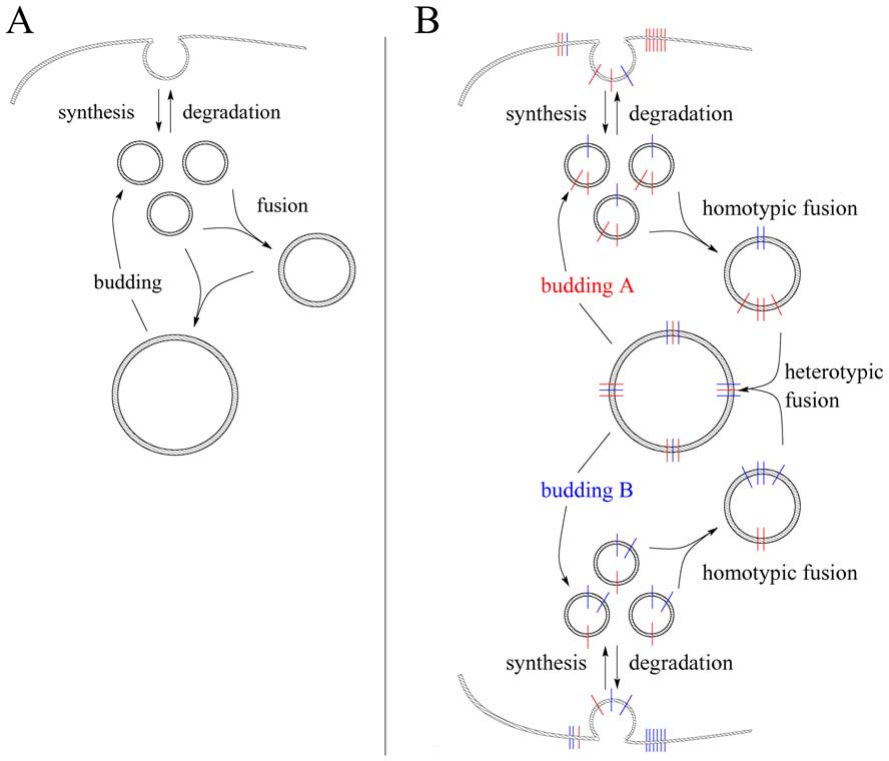
Organelle formation. A: At a point of origin (e.g. cell membrane) unit vesicles emerge and may be exported again or fuse with each other. Organelles composed of at least two unit vesicles might bud off unit vesicles. B: At two distinct points of origin (e.g. cell membrane and ER) unit vesicles emerge enriched in A or B type fusogenic proteins. Correspondingly two budding processes with loading preferences on A or B type FPs may occur. Vesicle fusion is accomplished by complementary FP paring.

Following the mathematical concept of Becker and Döring originally developed for systems of self-reproducing supramolecular systems [14], [15], we describe the dynamics of our model vesicles by the following system of differential equations:
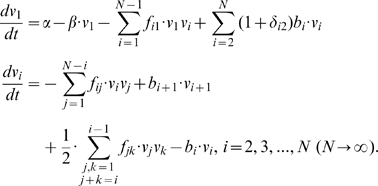
(1)Due to mass conservation in budding and fusion processes, the mass and surface area of vesicle 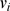 are 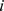-fold higher than the mass and surface area of the unit vesicle 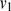 if we assume that the vesicle's lipid mass is concentrated in a thin surface layer. In Eqn (1) the factor 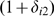 contains the Kronecker delta (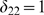 and 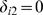 for 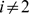) and takes into account that 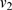-vesicles decay into two unit vesicles. Fig. 1A shows a scheme of the considered system where unit vesicles appear at a point of origin, e.g. they may be *de novo* generated during endocytosis at the cell membrane or at the endomembrane system. Table 1 lists variables, rates and parameters used in the L-model.

**Table 1:** L-model.

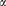	Generation rate of unit size vesicles
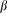	Parameter describing the degradation of unit size vesicles
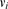	Concentration vesicles/organelles composed of a discrete number 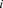 of unit vesicles with 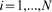
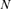	Number of unit vesicles the largest organelles are composed of
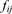	Fusion rate of two vesicles/organelles of size 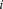 and 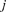 with 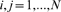
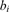	Budding rate of vesicles/organelles of size 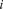 with 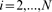
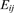	Activation energy needed to overcome the hydration repulsion between two approaching vesicles of size 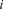 and 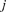 ( 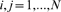 )
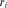	Radius of a spherical vesicle/organelle of size 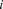 , with 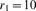 , 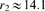 , 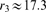 , …
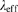	Phenomenological constant describing the effective proportional impact of the vesicle sizes on the energy barrier 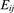
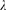	Decay length defined in [23] describing the repulsion of two vesicle membranes at a given distance. It depends on the lipid type or ion content of the aqueous environment
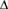	Appropriate chosen parameter defining the interaction cross section of two fusing vesicles (see Figure 9, Supplement S1)
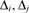	Two segments of 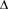 defined by the relative size of the two vesicles
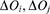	Two spherical caps determined by 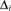 and 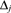
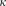	Parameter scaling the fusion rate to other rates
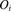	Surface of a spherical vesicle/Organelle of size 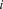 with 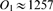
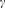	Parameter scaling the budding rate to other rates

Variables, rates and parameters relevant for the L-model described by Eqn (1) and in Supplement S1 and Figure 9. The lower rows are set apart and list quantities specified by resolving the fusion and budding rate in more detail.

#### Fusion rate 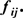


The mathematical expression for the fusion rate has to take into account that the fusion probability decreases with increasing size of the vesicles. This is accounted for by the so-called hydration repulsion [16]–[20] arising from water molecules that in aqueous electrolytes tend to tightly bind to ionised surfaces like membranes. To enable a direct contact between the membranes of two vesicles 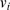 and 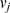 approaching each other, this surface shell of water molecules has to be removed. The necessary activation energy, 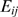, increases with increasing size of the fusing vesicles [21], [22].

As shown in Supplement S1 and Figure 9, one can approximately put 
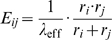
. This expression takes into account that the water-covered interaction surface between colliding vesicles becomes larger with increasing vesicle size implying that larger energies are required to strip off the water shell prior to fusion. However, if a small vesicle fuses with a large organelle (e.g. 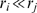), then only the membrane size of the small vesicle must be accounted for by considering the energy to strip off water molecules.

The parameter 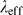 takes into account factors influencing the energy barrier as, for example, the specific head groups of membrane lipids or the ion composition of the surrounding aqueous phase. The latter effect plays a crucial role in many fusion processes where ions enhance fusion by lowering the energy barrier [23]. This effect is due to the sequestering of water molecules from charged vesicle surfaces by ions.

Using the well-known Arrhenius equation to link the activation energy barrier with the reaction rate, the expression for the fusion rate reads
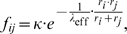
(2)where the factor 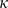 scales the absolute fusion rate to the rates of the other processes.

Additionally to an exponential decrease of the fusion likelihood with growing vesicle radii, an antagonistic increase of 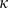 with a growing cross section might be accounted for, leading supposably to an increase of the fusion likelihood with the square of the radii. However, this would yield a comparable moderate influence on the fusion rate in particular if one keeps in mind that vesicles are transported to their destinations via the cytoskeleton which makes the probability of encountering of two vesicles little dependent on the radii. Moreover, we aimed for a simple conceptual and phenomenological description of the size dependence of the fusion rate to keep the model complexity and the computational effort low and therefore neglected the contribution of a cross section.

#### Budding rate 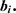


We assume that only unit vesicles are pinched off, i.e. 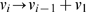. The budding rate is set proportional to the size of the sending vesicle, i.e. 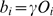.

With the above specifications of the fusion and budding rates, the infinite equation system (1) was truncated at a finite 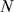 and then numerically integrated. The truncation threshold 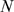 was chosen large enough to assure that the simulation results were not changing when the calculation was repeated at 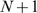. In general, our approach is not restricted to that specific choice of parameters and even more complex fusion and budding mechanisms are conceivable.

**Figure 2:**
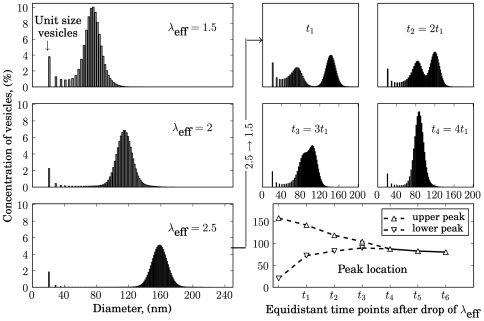
L-model: Steady state distributions of organelles and transient evolution. Left panels: steady state distribution of vesicles and organelles. Rate constants are: 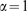, 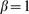, 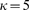, 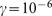, 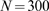, 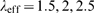. Right panels: temporal evolution of organelle sizes after decrease of 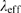 from 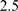 to 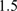. Transiently, a pool of smaller organelles emerges in parallel to the pool of larger ones.

Fig. 2 shows the stationary size distribution of vesicles at varying values of the parameter 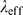. The peak of the size distribution defines the average size of an “organelle”. According to Eqn (2), increasing the value of 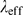 diminishes the inhibitory effect of the repulsion force on the fusion process and thus increases the average size of the lipid organelle. Experimentally, an increase of 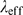 can be easily achieved by addition of Ca ions weakening the interaction of water molecules with the membrane surface. The right-shift of the size distribution shown in Fig. 2 (left panels) has also been observed in suspensions of lipid vesicles upon addition of CaCl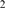 [24].

The four upper right panels show some intermediate steps in the temporary evolution of organelle sizes induced by a decrease of the parameter 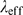 from 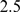 to 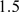. During the transition from the initial size distribution peaking at an organelle diameter of 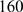 nm to the new steady-state size distribution peaking at an organelle diameter of 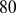 nm a smaller peak appears temporarily. At time-point 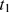 this small peak resides around 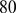 nm. It originates from a decrease of fusion activity of unit vesicles with large organelles in combination with a continuing budding activity of these large organelles as before. This results in a short-lived abundance of unit vesicles which subsequently fuse among each other forming transiently a second peak around a smaller average organelle diameter. Both peaks than approach each other to merge into the new single size distribution as the larger organelles keep at shrinking in size via budding and the smaller ones find temporarily an excess of large fusion partners. In a last phase the merged single size distribution shifts as a whole gradually towards smaller sizes by ongoing budding events ultimately evolving to a steady-state identical to the distribution shown in the first panel. The lower right panel depicts more compactly the average organelle sizes of the two peaks traced over several equidistant time points (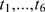) after drop of 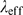.

### Formation of Organelles from Lipid Vesicles Containing Fusion Proteins (LP-Model)

The model was extended by endowing the lipid membrane of the vesicles with two different types of fusogenic proteins (FP), A and B. Fig. 1B shows a scheme of the considered system. A vesicle is now specified not only by its size index, but also by the number of FPs, i.e. 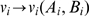 with 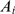 and 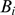 denoting the surface concentrations of FPs in a vesicle of size 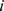. It has to be noted that in our model these fusogenic proteins are thought to represent molecular complexes composed of SNAREs, their activating Rabs and several other regulatory proteins all together needed to elicit fusion competence. Moreover, organelle specific resident proteins are conceived to be part of the FP complexes. The surface density of these fusion-competent FPs is considerably smaller than the surface density of SNAREs because it is restrained by that molecular component having the lowest concentration.

The fusion of two vesicles 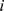 and 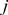 depends now additionally on the concentrations of A and B FPs present in both vesicles. Inspired by the SNARE hypothesis, we postulate that FPs resident in the membranes of different vesicles may bind each other under formation of a fusion complex provided that the vesicles are in sufficiently close proximity. FPs may fulfil two tasks. The formation of a fusion complex within the interface of two interacting vesicles facilitates the fusion process and additionally makes it more specific.

How to account for the first task may be easily illustrated mathematically by considering a novel factor 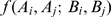 describing a positive function of FP concentrations. The function enters Eqn (2) as an additional contribution to the FP independent fusion:
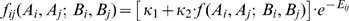
(3)Consistently, if no FPs were present it holds 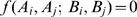 implying that Eqn (3) reduces to Eqn (2). Reformulation of Eqn (3) yields
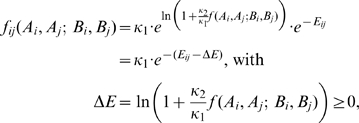
revealing that FPs act similarly to enzymes in metabolism which also lower the activation energy of a reaction. To simplify matters we will concentrate on the FP based contribution to the fusion process in the following and therefore assume its dominance over the basal fusion activity. With 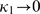 (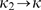) Eqn (3) retains a practical form:
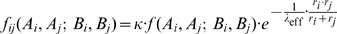
The second task of FPs concerns the fusion specificity and is less trivial. For this one has to specify 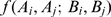 in (3). A simple assumption on how FPs enter the fusion rate is based on mass action kinetics. Here the fusion rate of two vesicles 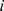 and 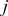 depends bi-linearly on the concentration of corresponding FP proteins in each vesicle:
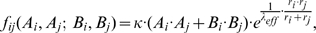
(4)where 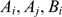 and 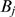 denote the concentrations of FPs in the vesicles 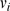 and 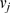. This approach is equivalent to a fusion rate used in [12]. As can be seen in Eqn. 4, type A FPs bind exclusively A and similarly type B FPs bind solely B proteins.

**Figure 3:**
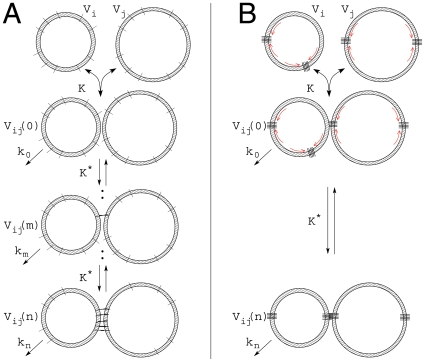
Types of cooperativity. Two approaching spherical vesicles with radii 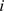 and 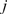 contain several FPs. (A) An elementary cooperativity is given if FPs successively form bridges with FPs of the same type on the opposite side establishing intermediates 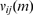 with equilibrium constants 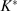. Each FP pair facilitates the subsequent pairings. (B) A Hill type cooperativity is imposed if a limited number of FPs assemble due to an intrinsic tendency or around hot-spot proteins (e.g. Rab effectors) prior to fusion. In any case fusion proceeds not before bridges comprising 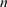 FPs have formed.

As demonstrated in several experimental studies [13], fusogenic proteins like SNAREs may form supramolecular aggregates within the membrane of a vesicle or organelle. These supramolecular aggregates rather than the individual fusion proteins are supposed to interact with each other under formation of a fusion complex of two vesicles in apposition. A similar effect is constituted by a lateral segregation before fusion, occurring at sites where membranes attach [25]. Given these findings more complex fusion kinetics are conceivable. Fig. 3A shows a situation where the FPs present at the surface of two colliding vesicles successively form molecular bridges producing intermediates 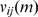 differing in the number of FP pairings (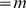) established. Each FP pair is supposed to tighten the inter-vesicle connection which imposes a certain degree of cooperativity on the fusion procedure. The maximal number of such bridges is assumed to be 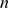 (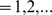) defined by the energy barrier to overcome for successful fusion. An appropriate fusion rate based on the successive formation of bridges as shown in Fig. 3A reads
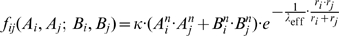
(5)A mathematical motivation of Eqn (5) based on detailed molecular mechanisms is given in Supplement S1.

Fig. 3B depicts a more advanced type of cooperativity arising if FP aggregates may assemble prior to fusion [13], [25]. Conceivable is that a limited number of FPs may assemble due to an intrinsic tendency within restricted membrane areas or around regulatory hot-spot proteins (e.g. Rab effectors). These intra-membrane aggregates will certainly give rise to saturation effects depending e.g. on the capacity of the interaction surface in which the assembling of FPs may take place or on the availability of regulatory proteins (e.g. specific Rab effectors). Subsequent to aggregation FPs will pair with opposite FPs of the same type. Describing the number of intra-membrane supramolecular aggregates composed of 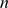 (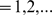) copies of a FP of type A or B by a Hill-equation and putting the fusion rate proportional to the concentrations of the aggregates present in the two colliding vesicles, the extended expression for the fusion rate now reads
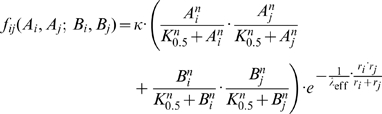
(6)The parameter 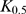 denotes a half-saturation constant for the formation of intra-membrane aggregates which we fixed at 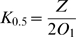. In conclusion, for Eqn (6) two factors are assumed to be probably independent from each other and operate in concert: An intra-membrane segregation leads to a local enrichment of cognate and/or de-enrichment of non-cognate FPs, and a pairing preference for cognate FPs in the trans-configuration [25].

To keep the model variants as simple as possible Eqn (4–6) take into account only homotypic pairing of FPs. In the following we will compare the three LP-model variants represented by different fusion kinetics.

Next, we have also to specify in which proportion the two different FPs are being transferred to the *de novo* generated unit vesicles as well as to the released unit vesicles during a budding process from larger organelles [26]. The rate equations for these processes are motivated by the fact that interaction with specific coat proteins enables to disproportionate proteins in the membrane of produced vesicles. We distinguish between an A-type and a B-type unit vesicle generation process, each one enriching the generated vesicle either in A or B. The *de novo* formation of unit vesicles comes now in two forms: 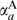 and 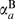, where the upper index indicates the two basic generation types and the lower index symbolises the number of A type FPs loaded into the generated vesicle.

Similarly, we define an A-type and a B-type budding process, each one enriching a budded unit vesicle from an organelle either in A or B. One may identify these two types of processes as coatA and coatB initiated budding events [12]. Budding depends additionally on the number of A and B type FPs present in the organelle from which the vesicle buds off. Thus, the single rate constant 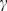 is replaced by two matrices 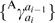 and 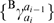 describing the associated budding rate constants. They specify the probabilities that from an organelle of size 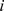 with 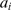 FPs of type A a vesicle buds off leaving behind an organelle of size 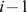 with 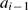 FPs of type A. A recursion formula for their determination is given in Supplement S1.

**Figure 4:**
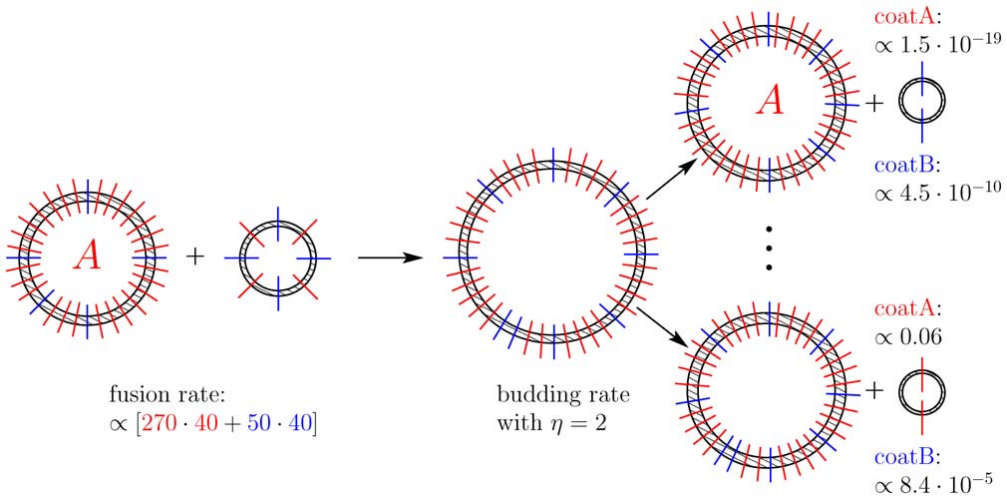
Fusion and budding. A fusion process between an organelle with A/B-composition 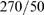 and a smaller vesicle with 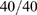 leads to a hybrid organelle which may bud off differently enriched unit vesicles. A colored line represents 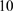 FPs. As examples, the matrix entries for the budding of fully enriched vesicles are given as 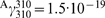, 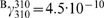, 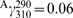 and 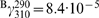, calculated with 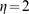. The highest budding probability for a coatA budding event is 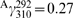 and for a coatB event 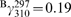.

A well defined organelle with proper biochemical identity must be assembled by at least 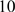 unit vesicles, which guarantees that the organelle size is located reasonably well within the the second peak of the stationary size distribution (see Fig. 2). Moreover, it must contain at least 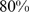 of either A or B type FPs to be considered as a characteristic organelle equipped with predominantly one type of marker. Fig. 4 depicts a possible scenario where an A type organelle fuses (bi-linear kinetics) with a smaller entity having no characteristic identity and is therefore regarded as a “hybrid” vesicle. The result is again a hybrid organelle which may bud off 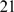 differently loaded unit vesicles. Each may emerge in two ways according to the basic budding types, based on coatA and coatB proteins. For the two fully enriched unit vesicles the budding matrix entries are given. In case a fully B-enriched vesicle is budded off the sending organelle receives an A type identity.

The complete equation system is described as follows:
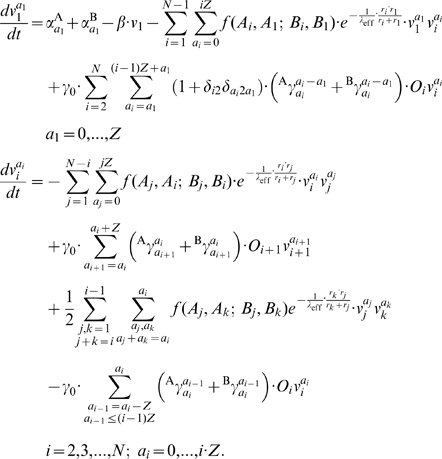
(7)An organelle of size 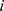 may contain between 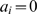 and 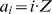 FPs of type A. Moreover, at any time it holds 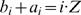. Table 2 lists parameters and variables specifically relevant for the LP-model.

**Table 2:** LP-model.

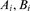	Concentrations of A and B type FPs in vesicles of size 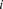 .
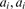	Number of A and B type FPs in vesicles of size 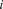 .
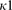	Scaling parameter for a basal, i.e. FP independent fusion rate
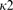	Scaling parameter for the FP dependent fusion rate
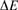	By this quantity the activation energy can be reduced due to FPs
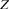	Total number of FPs in a unit vesicle (binding sites for FPs) [38]
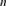	Degree of cooperativity: ultimate number of FP bridges for a successful fusion (model variant with FP bridges/pairs) or number of participants within aggregates of FPs
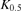	Half-saturation constant for the formation of intra-membrane aggregates
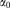	Parameter scaling the fusion rates in relation to other rates
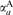	CoatA (loading preference of A type FPs) unit vesicle generation rates. The loaded number of A type FPs is 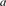 , with 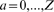
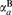	CoatB (loading preference of B type FPs) unit vesicle generation rates. The loaded number of B type FPs is 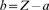 , with 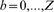
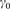	Parameter scaling the budding rates in relation to other rates
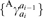	Biased budding matrix (loading preference for A type FPs) describing the loading of 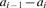 FPs to a unit size vesicle which buds off from an organelle of size 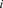 ( 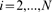 ) and FP content 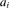
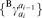	Biased budding matrix (loading preference for B type FPs) describing the loading of 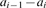 FPs to a unit size vesicle which buds off from an organelle of size 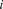 ( 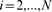 ) and FP content 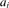
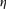	Parameter describing the degree of preference/bias with which a *de novo* generated vesicle is enriched/loaded with either A or B type FPs. The two budding matrices are calculated on the basis of 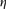 , too (for details see Supplement S1)
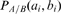	Probabilities of loading a single A or B type FP, respectively, to a coatA vesicle budding off from an organelle of size 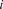 with a content of 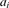 and 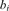 FPs. Two similar probabilities of loading a single A or B type FP to a coatB unit vesicle may be defined
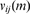	Concentration of intermediates formed by two attached vesicles/organelles of size 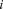 and 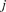 and 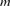 pairs of FPs established for maintenance (see Fig. 3A)
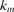	individual first order fusion rate constants describing (mostly premature) fusion events of intermediates ( 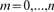 )
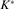	Equilibrium constant describing the equilibria between the fusion intermediates
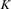	Association constant (vesicle size dependent) describing the equilibrium between the unattached vesicles and 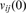
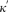	Lumped fusion parameter: 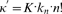

Rates and parameters relevant for the LP-model described by Eqn (7), in Supplement S1 and Fig. 3A. Two sets of rows which are set apart list quantities specified by resolving budding and fusion rates in molecular detail.

For a proper comparability of the three fusion kinetics with respect to the distribution of FPs within organelles we go after a similar average organelle size in each case. To ensure this we must employ if necessary an additional scaling because the impact of FPs on the fusion frequency varies substantially for the different kinetics. As a suitable reference situation in which the different kinetics are to adjusted we chose the case of fusion of two vesicles each containing equal concentrations of A and B: 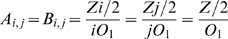. In case of a Hill type kinetics (6) the fusion frequency is:
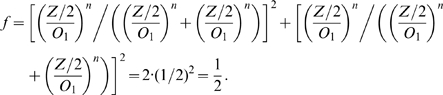
For non-Hill type fusion frequencies equal concentrations of A and B yield
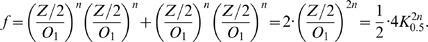
(8)For a similar average organelle size in each case we aim for the same order of magnitude in the fusion frequency by adapting the units. We therefore employ the scaling 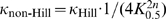 in all calculations. Specifically 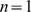 holds for bi-linear mass action kinetics.

**Figure 5:**
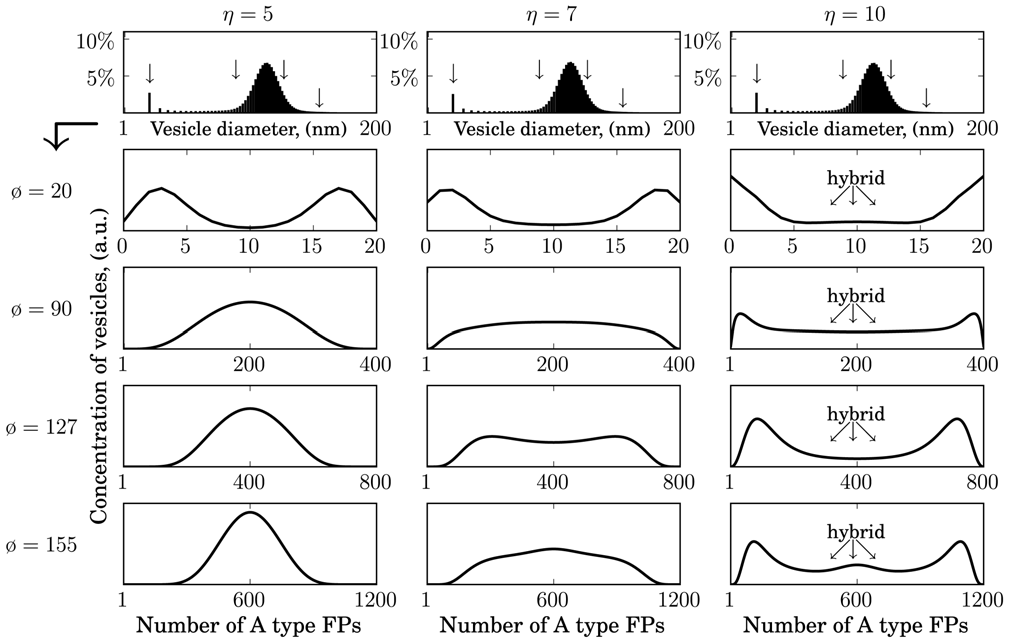
LP-model: Steady state distributions of vesicles/organelles assembled via a fusion machinery based on bi-linear mass action kinetics. Shown are size distributions for 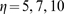. The panels below expand the vesicles of certain sizes, marked by arrows in the size distributions, due to their A type FP content. For clarification in case of 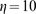 vesicles with unspecific identity, i.e. approximately equal numbers of A and B type FPs, are denoted as “hybrid”. Parameters: 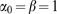, 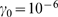, 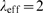, 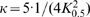.

**Figure 6:**
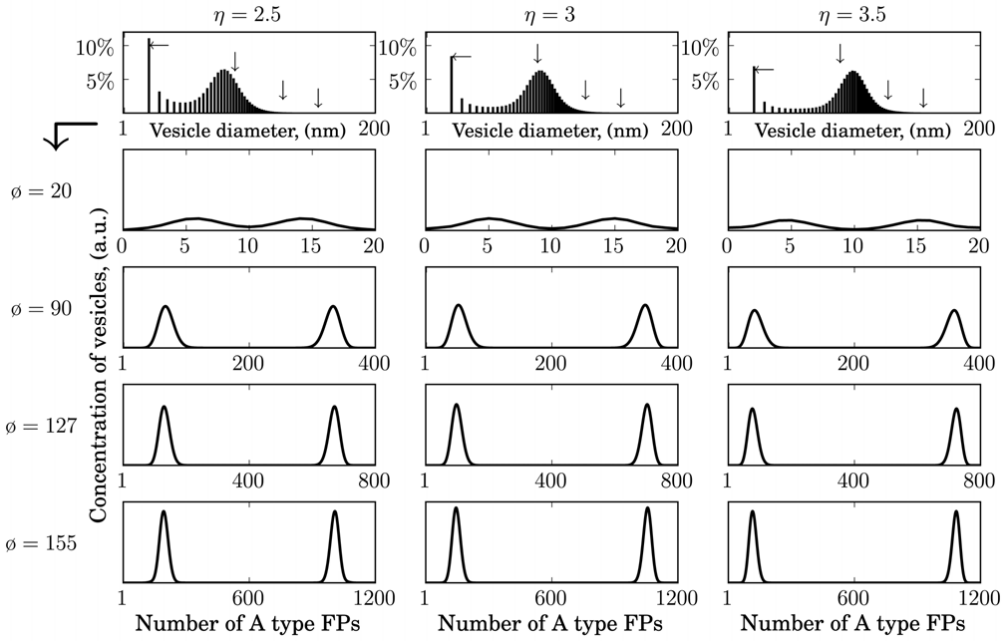
LP-model: Steady state distributions of organelles assembled by fusion based on hyper-linear kinetics (successive paring). Enrichment strengths: 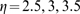. Other parameters as in Fig. 3 except 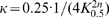 and 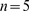.

**Figure 7:**
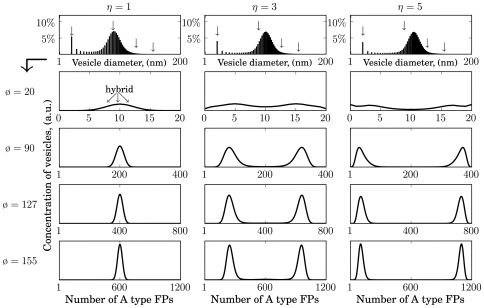
LP-model: Steady state distributions of organelles assembled by fusion based on hyper-linear Hill kinetics. Shown are size distributions and FP compositions for 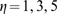. Parameters as in Fig. 4 except 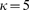 and 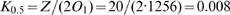.

We performed extensive numerical simulations of the model to study the impact of the enrichment strength 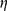 and the type of fusion kinetics on the stationary size distribution of vesicles as well as on the distribution of FPs across vesicles of different size. We put the total number of FPs in a unit vesicle to 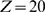 and the truncation threshold to N = 100. As with the simple L-model, it was possible to generate organelle-like size distributions (upper panels in Fig. 5, 6, 7). Fig. 5 shows the size distributions for three different enrichment factors: 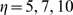 in the case of bi-linear mass action kinetics. The four panels thereunder depict the distribution of the fusogenic protein A (identical for type B) in organelles of different sizes, i.e. surface areas (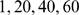) corresponding to certain diameters (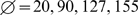). They are indicated at the left margin and marked by arrows in the upper panel. Obviously, towards larger organelles the distributions approach normal distributions, revealing that the most abundant organelles are those which contain equal numbers of A and B type FPs. Intriguingly, in the case of mass action fusion kinetics, it was not possible to generate vesicles or quasi-organelles that exhibited a bi-modal distribution of one type of FPs. The fact that barely organelles appear differing significantly in the relative share of FPs was not independent of the choice of the value of the enrichment factor and the average organelle size (ø
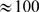
nm). A strong enrichment (e.g. 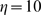) indeed achieves a certain degree of disproportionation of FPs in unit vesicles but still many “hybrid organelles” [7] containing about 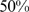 of both FPs are visible. More importantly however, the larger the organelles (late endosomes: ø
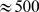
nm, [27]) the less a bi-linear fusion kinetics is able to prevent such a complete mixing of FPs. Although it is computational to expensive to calculate a system with such a large average organelle size it is clear that in such a system a high fraction of organelles without biochemical identity would be dominant. The reason for the observed phenomenon is that the disproportionation of A- and B proteins accomplished during the enrichment of unit vesicles is abolished through mixing during fusion processes if the interaction of the FPs obeys linear mass action kinetics. This effect is the more pronounced the larger the organelles are.

For comparison, organelles arising from a fusion mechanism based on cooperativity among FPs are depicted in Fig. 6 (successive paring) and Fig. 7 (Hill kinetics based on fusogenic aggregates). Fig. 6 shows the results based on the scenario given in Fig. 3A The corresponding fusion rate is described by Eqn (5). Strikingly the range of 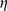-values (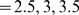) must be chosen more closer to achieve similar average organelle sizes in all three cases. This emphasises the high enrichment-sensitivity of a vesicular transport system which is based on cooperative fusion kinetics induced purely by successive paring. The value for 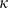 was adjusted by a necessary unit-scaling due to Eqn (8). An additional lowering of 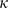 (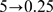) accounts for the differences in 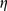-values compared to Fig. 5. These adjustments in the 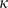-value enable proper aligned size distributions.

For 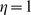 again no organelles with biochemical identity may emerge (data not shown). Thus, the enrichment of unit vesicles with one or the other type of marker proteins remains a necessary prerequisite for the maintenance of biochemical identity of an organelle [12]. However, already for relative mild enrichment strengths one finds proper bi-modal distributions of organelles with respect to their FP content: Organelles with the same size subdivide into two sub-populations containing either predominantly type A or type B FPs.

Fig. 7 shows the results based on the scenario given in Fig. 3B. The corresponding fusion rate (Hill type) is described by Eqn (6). For a Hill type cooperative fusion it was possible to calculate steady states for a wider range of 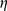-values yielding similar average organelle sizes. This implies that a vesicular transport system based on such a fusion machinery (e.g. pre-formed aggregates) is much less sensitive to the enrichment strength of budding vesicles compared to a hyper-linear cooperative fusion kinetics based on successive FP paring (cf. Fig. 6). However, similar to the case of the processive paring for 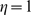 no organelles with biochemical identity may emerge, but again for relative mild enrichment strengths one finds bi-modal distributions of organelles with respect to their FP content. It is important to note, that this trend to form non-identical organelles enriched in A- or B-type FPs becomes more pronounced with increasing organelle sizes.

**Figure 8:**
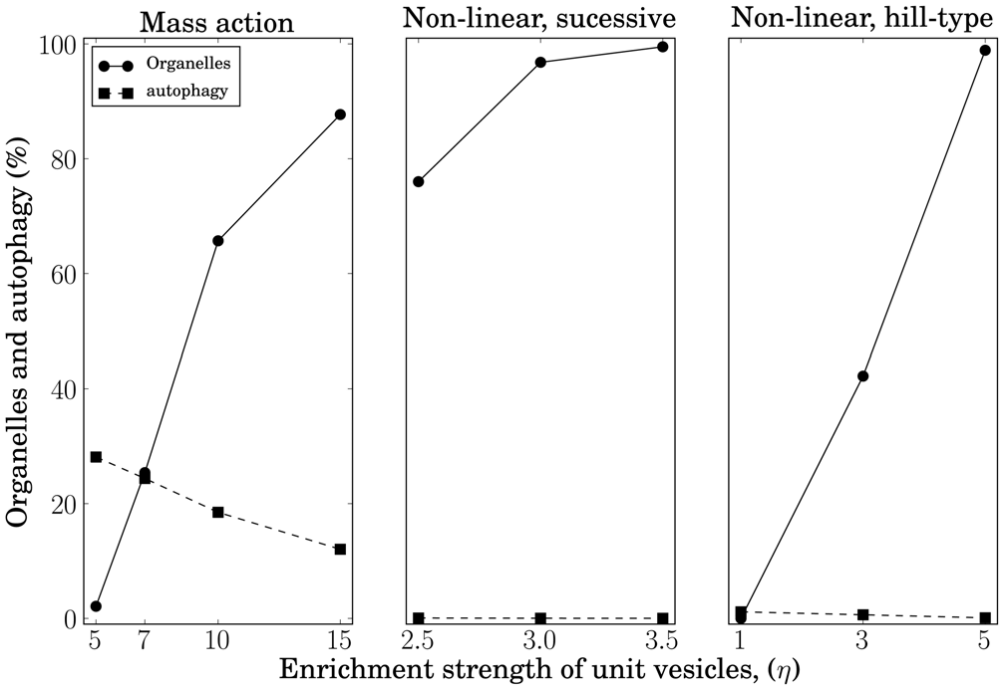
Impact of fusion kinetics and enrichment strength 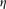 towards identity and autophagy. Depicted are the number of proper organelles related to vesicles of comparable size (squared marker). Additionally the number of autophagy-like events in relation to all organelle fusion processes are shown (circles).

Fig. 8 gives a compact overview of how the enrichment strength influences the fraction of emerging organelles (straight line with filled circles) assuming three different fusion kinetics.

**Figure 9:**
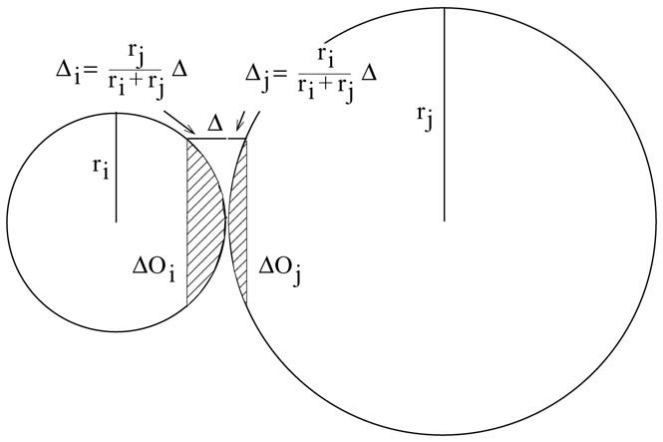
The hydration repulsion of two approaching vesicles. Water molecules attached to the charged membranes must be removed before a successful fusion. We assume monolayers of water molecules establishing an energy barrier 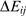 which is to overcome. Only the molecules in the shape of two spherical caps (shaded) with surfaces 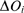 and 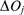 must be removed.

A second revealing entity is the amount of fusion events between well defined organelles differing in their biochemical identity. In this way one organelle type is absorbed by another and may not retain its identity. Such events may be interpreted as autophagy-like processes involved in selective organelle degradation. The square markers (dashed line) in Fig. 8 indicate the total sum of fusion fluxes wherever two distinct organelles fuse in relation to the total sum of fusion fluxes of any two organelles. One finds that the more pronounced their identities (increasing enrichment) the less likely distinct organelles fuse, i.e. the fewer autophagy-like processes occur. In case of cooperative fusion this effect is potentiated.

Our model is conceptual and qualitative, rather than quantitative, because many of the parameters, such as the fusion and budding constants are only known very approximately. As showcases we presented simulation results for selected parameter values rather than systematically analysed all possible scopes of influencing factors. However, the characteristic distributions of organelle sizes and FPs within them do not only exist for the chosen values of the parameters, but rather show up for a wide range of values. Adopting the concept of Metabolic Control Theory [28] further theoretical studies could be directed towards quantifying in a more systematic manner the influence of the various model parameters (e.g. enrichment strength, half-saturation constant 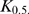, degree of cooperativity 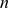, etc.) on organelle properties such as size distribution or abundance of marker proteins.

## Discussion

Vesicles and organelles contain many proteins which support the fusion process. Among them are tethering factors, adaptors, GTPases and SNARE proteins. In particular corresponding SNARE proteins [29] are thought to accelerate vesicle fusion processes. Since models should be as simple as possible but not simpler (Albert Einstein) we developed a minimal model to describe the *de novo formation* of biochemically diverse organelles. It includes vesicle generation and destruction, fusion and budding processes and two types of fusogenic proteins (FP) playing the role of identity giving marker proteins.

The simulations suggest that the interaction of homotypic FPs governed by a bi-linear mass action kinetics is not sufficient to counteract the permanent mixing of membrane components during fusion and thus does not allow the formation of organelles with sufficiently unequal protein content. This theoretical finding is supported by experiments demonstrating that the energy from a single SNARE complex might not be sufficient to overcome the full fusion energy barrier [25], [30]. Rather, to accomplish fusion, multiple copies of SNARE complexes must work in cooperative manner [13] by forming supramolecular structures prior to the actual fusion event. Indeed, introducing hyper-linear fusion kinetics our model simulations predict the occurrence of organelles with distinct protein contents.

A cooperative fusion machinery ensures that two vesicles having low numbers of non-corresponding FPs are very unlikely to fuse. The simulations also suggest that the separation of organelles into biochemically distinct sub-populations (here: of A- or B-type) becomes more and more pronounced with increasing organelle size and thus should be almost perfect for real cellular organelles as lysosomes or peroxisomes. It has to be noted that this finding is independent on the initial conditions, i.e. even if the system starts with unit vesicles strongly enriched in A and B FPs, bi-linear mass action kinetics is not sufficient to generate proper organelles with segregated FPs.

Our simulations have also shown that besides a cooperative fusion kinetics a certain degree of enrichment of budding vesicles with specific types of proteins must be guaranteed. Such a separation of proteins destined for transport from those destined to remain in the donor vesicle can be accomplished by coat proteins involved in the budding process. Experiments with synthetic phospholipid liposomes have demonstrated that assemblies of cytosolic coat proteins are able to direct specific v-SNAREs (Sec22p) into budding COPII vesicles [31].

Intriguingly, in case of a cooperative fusion kinetics based on successive FP pairing the impact of disproportionation of FPs during the budding process (enrichment strength) on the average organelle size was much stronger than in case of a Hill based fusion kinetics (pre-formed aggregates). To discriminate between these two conceivable modes of cooperativity further structural studies are needed. At the moment, spontaneous self-aggregation of FPs seems very likely in the light of recent experiments demonstrating in detail how cluster formation of syntaxin 1 and syntaxin 4 (Q-SNARE proteins participating in exocytosis) primarily involving the SNARE motif proceed [32].

Our finding that cooperative homotypic fusion appears to be more efficient in providing biochemically distinct organelles than disproportionating FPs during budding is in concordance with the observation that the diversity of proteins involved in vesicle fusion (e.g. SNAREs and Rabs) is much higher than that of rather unspecific coat proteins [33]. A similar asymmetry between the molecular complexity of antagonistic processes is also observed in cellular signal transduction where the repertoire of specific protein kinases (usually activating their protein target) is much larger than the number of relatively unspecific phosphatases [34].

In an evolutionary context, it appears that primordial cell types getting along with relative small organelles may have organised distinct biochemical identities of them exclusively by means of a budding machinery enabling selective enrichment of membrane proteins in released vesicles. However later, when cells became more complex and organelles larger, it was essential to develop additionally a fusion mechanism based on hyper-linear cooperativity among the specific marker proteins. This transition was perhaps accompanied by a drop in the selection pressure towards the enrichment strength such that one possibly encounters nowadays a strong cooperativity in the fusion machinery in combination with weak budding enrichments of marker proteins.

A further increase in complexity during evolution could have been to replace a simple type of cooperativity (pure successive paring) by an elaborated FP interplay based on already pre-formed aggregates of fusion proteins. Such a design also opens up a potential mechanism for organelle maturation and identity switching (e.g. early to late endosome) because the activation of fusion proteins and thus the critical concentration of active fusion proteins needed for the clustering process (in our model represented by the parameter 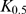) can be linked to signalling pathways [35] or lumenal acidification processes both playing a major role in organelle maturation.

A following extended model will include explicitly the membrane systems from where unit vesicles are *de novo* generated: the plasma membrane and the endomembrane system. Moreover, fusion and budding machineries will be resolved in more detail by considering Rabs, SNAREs, cargo proteins and lipid components seperately. Due to the high complexity of this model a stochastic approach is needed [37].

## Materials and Methods

We provide a software tool specifically designed for modelling large scale organelle formation systems by means of vesicular transport. The software is available on our homepage: http://www.charite.de/sysbio/people/binder/download/.

## Supporting Information

Supplement S1 The supplementary information describes molecular details of the fusion and budding machineries. It comprises four parts: 1. Vesicle size dependence of the fusion rate constant 2. Hyper-linear fusion rates 3. De novo generation of unit size vesicles 4. Budding matrices
(0.06 MB PDF)
