# Supplementary material for: A Conceptual Mathematical Model of the Dynamic Self-Organisation of Distinct Cellular Organelles
Source: PLoS One. 2009 Dec 30;4(12):e8295. doi: 10.1371/journal.pone.0008295 (PMC2795802; doi:10.1371/journal.pone.0008295)
Supplement: Supplement S1 — The supplementary information describes molecular details of the fusion and budding machineries. It comprises four parts: 1. Vesicle size dependence of the fusion rate constant 2. Hyper-linear fusion rates 3. De novo generation of unit size vesicles 4. Budding matrices (0.06 MB PDF) [file pone.0008295.s001.pdf]

Supplemental material

Contents

1 **Vesicle size dependence of the fusion rate constant** 1

2 **Hyper-linear fusion rates** 1

3 ***De novo* generation of unit size vesicles** 2

4 **Budding matrices $\{{}^{\mathbf{A}}\gamma_{a_i}^{a_{i-1}}\}$ and $\{{}^{\mathbf{B}}\gamma_{a_i}^{a_{i-1}}\}$** 2

1 Vesicle size dependence of the fusion rate constant

Due to the hydration repulsion water molecules attached to the charged membranes must be removed to overcome the energy barrier ΔE_{ij} of two fusing vesicles i and j. For the shaded spherical caps (ΔO_i and ΔO_j) in Fig. 9 which cover these molecules one must chose an appropriate parameter Δ defining the necessary interaction cross section to be considered. In the simplest case one may expect $\Delta E_{ij} \propto (\Delta O_i + \Delta O_j) = 2\pi r_i \Delta_i + 2\pi r_j \Delta_j$. For the two segments of Δ, named Δ_i and Δ_j (see Fig. 9), a reasonable approximation is given by $\Delta_i = r_j/(r_i + r_j) \cdot \Delta$ and $\Delta_j = r_i/(r_i + r_j) \cdot \Delta$ which gives a compact form of the energy barrier entering Eq. (2):

$$\Delta E_{ij} = \frac{2\pi\Delta}{\lambda} \cdot \frac{r_i \cdot r_j}{r_i + r_j} = \frac{1}{\lambda_{\text{eff}}} \cdot \frac{r_i \cdot r_j}{r_i + r_j}, \text{ with } \lambda_{\text{eff}} = \lambda/(2\pi\Delta),$$

playing the role of an phenomenological proportionality factor.

2 Hyper-linear fusion rates

The total fusion rate f_{ij} of two vesicles i and j is composed of individual fusion rates:

$$f_{ij} = f_{ij}(0) + f_{ij}(1) +, ..., + f_{ij}(n) = k_0 \cdot v_{ij}(0) + k_1 \cdot v_{ij}(1) +, ..., + k_n \cdot v_{ij}(n), \quad (1)$$

where $v_{ij}(m)$ describes the concentration of intermediates with m FP pairs already formed (see Fig. 3A). We assume rapid equilibria between successive intermediates $v_{ij}(m)$ and $v_{ij}(m+1)$:

$$v_{ij}(m+1) = \frac{(n-m)(n-m)}{m+1} \cdot K^* v_{ij}(m),$$

where the factor $(n-m)(n-m)$ describes the combinatorial multiplicity of pairing options of one additional FP pair and $(m+1)$ accounts for the possible unpairing processes. It can be easily shown that

$$v_{ij}(m) = \binom{n}{m}\binom{n}{m} \cdot m! \cdot (K^*)^m \cdot v_{ij}(0),$$

with $v_{ij}(0) = K \cdot v_i v_j$. Eq. (1) can then be rewritten in a compact form:

$$f_{ij} = K \cdot v_i v_j \sum_{m=0}^{n} k_m \binom{n}{m} \binom{n}{m} \cdot m! \cdot (K^*)^m.$$

We assume that a successful fusion can be accomplished primarily if at least n FP pairings could be established (overcome of energy barrier), i.e. $k_0, ...k_m, ..., k_{n-1} \ll k_n$. The accelerating effect of SNARE pairing is accounted for by the association constant K* describing the stabilisation of the fusion intermediate by SNARE pairing. If no pre-formation of aggregates is assumed K^* will certainly depend linearly on the concentration of total available FPs: $K^* \propto A_i A_j$:

$$f_{ij} = K \cdot v_i v_j \cdot k_n \cdot n! \cdot A_i^n A_j^n = \kappa' \cdot A_i^n A_j^n \cdot v_i v_j,$$

with $\kappa' = K \cdot k_n \cdot n!$. In the case of two independent FPs A and B, one retrieves

$$f_{ij} = \kappa' \cdot (A_i^n \cdot A_j^n + B_i^n \cdot B_j^n) \cdot v_i v_j,$$

where κ' accounts for the vesicle size dependent association constant K (repulsive hydration force). A specific assumption of this dependency yields Eq. (5).

In the scenario of pre-forming aggregates (see Fig. 3B) a concerted transition from $v_{ij}(0)$ to $v_{ij}(n)$ is assumed to proceed by a single step. In this case K^* depends on the number of aggregates instead of single FPs giving rise to a Hill type cooperativity as described in Eq. (6).

3 *De novo* generation of unit size vesicles

The enrichment of unit vesicles with either A or B type FPs results in coatA or coatB unit vesicles, respectively. For the initial *de novo* generation of such vesicles as well as for the budding process from organelles (see next section) we need a quantifier to describe the degree of biased loading during these two processes. We therefore introduce an enrichment factor $\eta > 1$ entering the two generation processes. As a consequence α_a^{A} and α_a^{B} are not two simple rates but rather two (symmetrical) binomial distributions with respect to a:

$$\begin{aligned}\alpha_a^{\mathrm{A}} &= \alpha_0 \binom{Z}{a} \cdot (\frac{\eta}{\eta+1})^a \cdot (\frac{1}{\eta+1})^{Z-a} \\ \alpha_a^{\mathrm{B}} &= \alpha_0 \binom{Z}{a} \cdot (\frac{1}{\eta+1})^a \cdot (\frac{\eta}{\eta+1})^{Z-a},\end{aligned}$$

with $a = 0, ..., Z$ describing the $Z+1$ possible numbers of loaded A type FPs. It has to be noted that if the number of A type FPs in a unit size vesicles is given, the number of B type FPs is determined as $b = Z - a$, where Z describes the total number of FPs in a unit size vesicle.

4 Budding matrices $\{^{\mathrm{A}}\gamma_{a_i}^{a_{i-1}}\}$ and $\{^{\mathrm{B}}\gamma_{a_i}^{a_{i-1}}\}$

The parameter $\eta > 1$ describes the increased loading affinity of an A type FP to its corresponding vesicle compared to the affinity of a B type FP to such a vesicle. Symmetrically, η characterises the preferential loading of coatB-based vesicles with B type FPs. As an example the $\{^{\mathrm{A}}\gamma_{a_i}^{a_{i-1}}\}$ matrix entries are calculated in the following. The probabilities P_A and P_B of loading a single A or B type FP, respectively, to a coatA vesicle which buds off from an organelle of size i with a_i FPs are given as:

$$\begin{aligned}P_A(a_i, b_i) &= \frac{\eta \cdot a_i}{\eta \cdot a_i + b_i} \\ P_B(a_i, b_i) &= \frac{b_i}{\eta \cdot a_i + b_i}\ .\end{aligned}$$

These elementary probabilities enter the recursion formula for ${}^{\mathrm{A}}\gamma_{a_i}(m,n)$ describing the successive loading of a vesicle with at present m FPs comprising $n \leq m$ FPs of type A:

$$\begin{aligned} {}^{\mathrm{A}}\gamma_{a_i}(m,n) = & {}^{\mathrm{A}}\gamma_{a_i}(m-1,n-1) \cdot P_A(a_i - n + 1, iZ - a_i + n - m) \\ & + {}^{\mathrm{A}}\gamma_{a_i}(m-1,n) \cdot P_B(a_i - n, iZ - a_i + n + 1 - m), \end{aligned}$$

for $m = 1, ..., Z, \;\; n = 0, ..., (a_i - a_{i-1})$ and ${}^{\mathrm{A}}\gamma_{a_i}(0,n) = 0 \; \forall n \neq 0, \;\; {}^{\mathrm{A}}\gamma_{a_i}(0,0) = 1$. The budding matrices in Eq. (7) are finally determined by

$${}^{\mathrm{A}}\gamma_{a_i}^{a_{i-1}} = {}^{\mathrm{A}}\gamma_{a_i}(Z, a_i - a_{i-1}).$$
